# Around the Hospitals

**Published:** 1894-08-25

**Authors:** 


### Around the Hospitals.

[Notice to the Managers of Institutions.—Special space is now reserved for the insertion of notices of hospital meetings and festivals. The Editor requests that all notices may reach The Hospital Office, 428, Strand, at least one week before the date of each meeting.]

**Sunderland Infirmary.**—More patients have been treated at this hospital during the past year—the hundredth of its existence—than in any previous year, every section of the infirmary showing an increase. The accounts show most satisfactory contributions from the working classes, which is in itself evidence that the work of the institution is appreciated by those for whom it chiefly exists. The expenditure has been £9,184 12s 8d., as against £9,169 19s. 2d. for the preceding year. This year being the centenary of the hospital, the committee suggest that it might be well celebrated by the extension of the Heatherdene Convalescent Home, in order to provide accommodation for male patients, and also by a special effort being made to add to the subscription list. The first of these objects has already met with encouraging support, and in response to the appeal of the miners and colliery workers in the district the Ecclesiastical Commissioners have promised the sum of £1,000. The importance of a convalescent home, and the great necessity of extending such an advantage to male patients is so obvious that no doubt the committee will receive the help they need with promptitude. Additions are also much needed to the administrative block of the infirmary, and as adequate accommodation for the nursing staff is one of the first essentials of a properly administered hospital, it must be hoped that means will shortly be found to carry these extensions into practice. An exhibition is to be held in Sunderland in the autumn, the promoters of which have generously offered the committee, in view of its being the centenary year, "certain interests in the undertaking in the hope that it may prove a pleasing and popular way of still further celebrating the event." Eleven probationers were accepted for training during 1893, 190 applications having been received for these vacancies. The sisters at this infirmary are supplied from the Deaconesses' Hospital at Tottenham, the nursing staff from probationers trained at the Infirmary. At the close of the yearly report the committee tender their warmest thanks to the medical and nursing staff, especially expressing appreciation of the services rendered by the Tottenham sisters. Cordial recognition is also given to the colliery owners for gifts of coal, and to Mr. W. B. Harrison for a daily supply of all the ice required.

